# Medically assisted integrated rehabilitation program for people with opioid dependence: a quasi-experimental evaluation using multi-criteria decision analysis

**DOI:** 10.1186/s12888-024-06416-5

**Published:** 2024-12-23

**Authors:** M. Kamrul Islam, Lars Thore Fadnes, Kjell Arne Johansson, Sabine Ruths, Maureen Rutten-van Mölken, Jan Erik Askildsen

**Affiliations:** 1 Department of Economics, University of Bergen, Postboks, Bergen, 7802, 5020 Norway; 2 Department of Health & Social Sciences, NORCE Norwegian Research Centre, Bergen, Norway; 3 Department of Addiction Medicine, Bergen Addiction Research, Haukeland University Hospital, Bergen, Norway; 4 Department of Global and Public Health, University of Bergen, Bergen, Norway; 5 School of Health Policy and Management, Erasmus University Rotterdam, Rotterdam, the Netherlands

**Keywords:** Opioid disorders, Medically assisted rehabilitation, Integrated care, Discrete choice experiment, Multi-criteria decision analysis, Quasi-experimental design

## Abstract

**Background:**

Opioid use disorders constitute a vast disease burden, need for comprehensive treatment, and substantial costs to individuals, families, and society. The multifaceted needs of people with opioid dependence call for integrated care. The study aims to assess the added value of an integrated medically assisted rehabilitation (MAR) program providing opioid agonist therapy for patients with opioid dependence as compared to the standard of care (SoC) in Norway.

**Methods:**

The intervention includes a comprehensive tertiary care integrated MAR program in Bergen. SoC is a much less intense primary care program in Oslo. 682 and 609 patients from Bergen, and 864 and 771 patients from Oslo were included in 2017 and 2019, respectively. A multi-criteria decision analysis (MCDA) framework was used where the relative preferences of the importance of the outcomes were obtained from a discrete choice experiment among five different stakeholder-groups. Seven outcomes related to health, well-being, experience of the care process, and cost were measured. The performance scores were measured in a study with a quasi-experimental design. Scores were analyzed using linear mixed methods. Performance scores for the outcomes were standardized and multiplied by their relative preferences to obtain the overall value scores in the MCDA.

**Results:**

We found similar value scores for both care delivery models regarding physical functioning, psychological well-being, social relationships & participation, enjoyment of life, and total costs. The Bergen-model scored higher on continuity of care (0.733 versus 0.680), while the SoC-model scored higher on person-centeredness (0.772 versus 0.635). Overall value scores were marginally in favor of the MAR-Bergen (0.708 versus 0.705 for patients).

**Conclusion:**

Acknowledging the significance of different life aspects emphasizes the need for integrated care at a specific level for people with opioid dependence. We conclude that the two highly effective treatment approaches produce promising outcomes in a challenging population and are quite similar. However, further research with more robust longitudinal data is needed.

**Supplementary Information:**

The online version contains supplementary material available at 10.1186/s12888-024-06416-5.

## Introduction

Opioid use disorders affect around 40 million people globally, causing more than 100,000 overdose deaths annually in addition to a 10-fold increase in mortality from other causes [1]. Among all illegal drugs, opioids represent the highest disease burden, with the highest need for treatment and contribute to substantial costs to individuals, families, and society [2]. People with opioid use disorders suffer from severe social marginalization and long-term impairments in most aspects of their lives [1]. Opioid dependence is a severe chronic relapsing disorder comprising a cluster of physiological, behavioral, and cognitive phenomena [1, 3].

Opioid substitution treatment reduces mortality by half [1, 4], and is a cornerstone in medically assisted rehabilitation (MAR) programs for opioid dependence. However, the multifaceted needs of people with opioid dependence with multiple chronic diseases call for an integrated care [5, 6], which brings a broad range of professionals from health and social care together to coordinate services. There is much progress yet to be made regarding an integrated treatment approach for people with opioid dependence. Overcoming the challenges to a holistic approach to opioid recovery is crucial to engage patients safely and comfortably, and to support sustained recovery.

It is widely argued that the value of integrated care goes beyond health-related quality of life. An integrated care approach for people with opioid dependence comprise medications aiming to prevent relapse and overdoses; group and individual counselling; physician visits for diagnostics, treatment, follow-up and medication management; mental health care; and psychiatric treatment for co-occurring disorders.

The multi criteria decision analysis (MCDA) framework is particularly suited to evaluate programs that aim to improve broader outcomes because it allows the combined measurement and weighting of multiple, sometimes conflicting, outcomes [7], for example including the “Triple Aim” for improving health care performance [8]. The “Triple Aim” comprises three interconnected aims: the first aims to enhance the health outcomes of entire populations, emphasizing prevention, health promotion, and addressing social determinants of health. The second aims to ensure that healthcare services are patient-centered, safe, effective, timely, efficient, and equitable. The third aims to decrease per capita healthcare spending while maintaining or improving the quality of care. In MCDA the ‘criteria’ (or outcomes) and multiple perspectives/preferences by different stakeholder groups can be assessed separately but also integrated into one overall value score by applying a weighting of outcomes [7].

Although MCDA has been recommended to support decision-making, its use remains limited [9]. Indeed, there remains a gap in the literature for the methodological implementation of MCDA for evaluating programs in health and social care, particularly programs targeting persons with problems in multiple life domains [9, 10]. Hence, this study is also an important contribution to the literature on applications of MCDA.

The main objective of this study is to investigate comparative effectiveness of two distinct medically assisted rehabilitation (MAR) programs–an integrated MAR-Bergen program as compared to the standard of care (SoC) in Norway. We hypothesized that MAR-Bergen is superior to SoC (considering MAR-Olso as the SoC). Using the MCDA framework, this study implements a comprehensive and transparent evaluation of MAR-Bergen against SoC.

## Methods

### Study population and data

MAR is a treatment program for opioid dependence using opioid substitution treatment as a cornerstone. In Bergen, the second largest city of Norway, a comprehensive tertiary-care integrated program for patients with opioids dependence has been provided through outpatient clinics since 2014 (referred to as MAR-Bergen). Oslo, the largest city of Norway, has to a larger degree opted for primary care-oriented program delivered through general practitioners (referred to as SoC). The two treatment models are well-suited for comparing two types of delivery models.

The study uses national data of MAR patients collected through the BrukerPlan register – a tool for mapping the extent and nature of the known substance abuse among service recipients. Professional staff does the mapping using their knowledge and assessments, and in that process, the users are color-coded (red/yellow/green) on the different functional assessments in eight areas of daily functioning. These include substance abuse, housing, work and meaningful activity, finances, physical health, mental health, social competence and networks. The tool is approved by the Norwegian Data Protection Authority for quality assurance, development and planning of services. Since 2011, the Norwegian Directorate of Health has supported efforts to get municipalities to use BrukerPlan as a mapping tool. The tool intends to provide municipalities and health trusts with a detailed and updated scenario of the users and their needs to assist the municipalities regarding prioritization and prediction of the future demand for health and care services [11]. Using the BrukerPlan mapping tool, most municipalities register data annually, however, some municipalities every other year. Data extraction was based on an anonymous patient serial number. We selected the years 2017 and 2019 for two reasons. First, in the BrukerPlan register, the mapping was conducted in both Bergen and Oslo (i.e., SoC) in 2017 and 2019. Second, for the weight elicitation study—to obtain weights—our discrete choice experiment (DCE) was conducted among Norwegian stakeholders between 2017 and 2019 (see below).

### Inclusion criteria

The inclusion criteria for a person to be added to the BrukerPlan register are (i) residents from the age of 16 onwards who have a professional-confirmed substance abuse problem; (ii) drug problems in this context are defined as problems that are seriously affecting daily functioning and relationships with others; (iii) the users are registered with municipal health and care services or state labor and welfare services and have received at least one service during the last 12 months.

In the BrukerPlan register there is a variable to identify whether patients are enrolled in MAR or not according to criteria given by ICD-10 people with opioid use disorders, (more specifically opioid dependence, F11.2). We used data for patients with confirmed participation in a MAR program, either in Bergen or Oslo.

### Intervention

The MAR-Bergen program is delivered by the Department of Addiction Medicine at Haukeland University Hospital in Bergen. In Bergen, opioid agonist therapy (OAT) outpatient clinics have been established in each district where the patients are followed up by health and social workers on a weekly basis with observed intake of the OAT medications. The core team of MAR-Bergen includes a physician, who is a specialist in addiction medicine, a psychologist, and a special counsellor/consultant (a nurse or social worker). Each special counsellor has a portfolio of patients, but there is also teamwork around each patient. In the team meetings, patients’ circumstances are discussed, and follow-up decisions are made. The involvement of different health and social professional groups aims at providing a holistic assessment of the patient with multimorbidity. Data were available for 682 patients participating in MAR-Bergen in 2017 and 609 patients in 2019. They constitute 91.9% and 87.9% of the total patients mapped through BrukerPlan in 2017 and 2019 respectively. However, we do not know whether all MAR-Bergen patients were included in the BrukerPlan data in 2017 and 2019.

### Comparison

The SoC program is delivered in collaboration between primary health care including general practitioners and the Department for Substance Use and Addiction Therapy at Oslo University Hospital. The SoC program treatment is initiated at the specialist specialist/hospital level. In most cases, after some week of stabilization the patient is transferred from specialist care to follow-up at the primary healthcare level with regular follow-up and prescriptions by the general practitioner and the social services in the local municipality where the patient resides. Specifically, the treatment is more decentralized and primary health care oriented than MAR-Bergen. The number of patients included from SoC was 864 in 2017 and 771 in 2019. They constitute 82.8% and 84.6% of the total patients receiving MAR in the two settings in 2017 and 2019, respectively. As with MAR-Bergen, we also do not know whether all SoC patients were included in the BrukerPlan data in 2017 and 2019.

### Study design

The study design includes a quasi-experimental impact evaluation, and the overall assessment was structured according to a novel MCDA framework. Anonymous patient serial number within the distinct BrukerPlan data prevented us from a longitudinal follow-up of distinct patients in 2017 and 2019. Following the approach by Deaton [12], we used the data to construct a pseudo-panel data set. In this approach, subjects sharing some common characteristics are grouped into cohorts (subgroups) [for detail see, 13–14]. Based on our selected patient’s time-invariant characteristics, namely, year of birth, gender, and location, at baseline (2017) we constructed a pseudo-longitudinal/panel data set which consists of 90 and 101 cohorts from MAR-Bergen and SoC respectively. Since some of the subgroups are not in 2019 (i.e., absence of one of the characteristics based on year of birth), the corresponding numbers of cohorts in the follow-up measurement (2019) are: 86 from MAR-Bergen and 91 from SoC.

### MCDA framework and decision context

The SELFIE (Sustainable integrated care models for multi-morbidity, delivery, financing and performance) MCDA framework was developed based on established guidelines and follows the seven recommended steps and implementation procedures described elsewhere [7, 15, 16]. Based on an earlier qualitative study, in the SELFIE framework, Patients, Partners and other informal caregivers, Professionals, Payers, and Policy makers are identified as relevant stakeholders [17–19].

### Outcomes and preferences

Although the outcome variables in the BrukerPlan register were designed for targeting persons with problems in multiple life domains, they do not match perfectly align with the SELFIE MCDA framework measures, thus we mapped the variables [15]. Appendix Table A1 provides a mapping of the outcome variables from BrukerPlan into their definitions in the SELFIE MCDA framework. Included outcomes in this study are physical functioning, psychological well-being, enjoyment of life, social relationships and participation, person-centeredness, continuity of care and total health, and social care costs. The questions that were asked to measure these outcomes are given in Table 1.

**Table 1:** Outcomes measured in MCDA

Criteria	Item (score)	Scale
Physical health	a) **Does not have physical health problems** with serious consequences for daily functioning. (3) b) **Has some physical health problems** that have serious consequences for daily functioning and / or for future health. (2) c) **Has extensive physical health problems** that have very serious consequences for the poor functioning and / or for future health condition. (1)	1–3 (best)
Mental health	a) Mental health condition **without any serious consequences** for functional level and / or relationship with others. (3) b) **Some functional impairment** due to mental health condition, fails to meet normal requirements for functioning towards friends, work / school, agreements, public transport, but reasonably takes care of their own daily chores and personal hygiene. (2) c) **Severe dysfunction** and failing forces, motivation and/or skills in relation to demands from the environment and for self-care (for example, daily chores and personal hygiene) because of the mental health condition. (1)	1–3 (best)
Social relationships & participation	Added two following characteristics: *Relationships with social networks*: a) **Has regular relationships** with social networks such as family, children, friends, voluntary organizations and/or working life. (3) b) **Has limited relationships** with social networks such as family, children, friends, voluntary organizations and/or working life. (2) c) **Has little or no contact** with social networks of all kinds, are isolated or have only marginal social relationships. (1) *Social functioning*: d) **Have good social functioning** to able to manage the daily chores. (3) e) **Limited social functioning** to able to a limited extent to take care of the daily chores in homes, shops and in contact with public offices. (2) f) **Has very poor functioning** in all types of contexts. (1)	2–6 (best)
Enjoyment of life	Whether have an engagement in meaningful activity e.g. in work, education, or other activities: a) with sufficient degree. (3) b) in some but not sufficient degree. (2) c) very little or no meaningful activity. (1)	1–3 (best)
Person Centeredness	Whether have an individual plan with patient or patient relatives: Yes = 1; No = 0	0–1 (best)
Continuity of care	Whether have a plan with general practitioner (GP) or health and social care service provider or plan with NAV or plan with specialist care provider or plan with PPTOT or pan with child welfare: Yes = 1; No = 0	0–1 (best)
Total health and social care cost	Health and social care cost were proxied by a *Living conditions index* which is a composite indicator containing eight living condition areas. The living condition areas constitute a score where the red score has the highest points. The index comprises the summed of the points from the eight living condition features	138–552 (worst)

The lack of data in the BrukerPlan register on patient use of healthcare services prevents quantifying the total health and social costs incurred. Instead, we used the living conditions index as proxy for total health and social care costs. The index, based on the eight living conditions areas, is scored using colors, with red indicating the highest points. When summed, the index ranges from 138 to 552 points (for detail, see [20]). The average index for recipients with substance abuse problems is 265.

**Fig. 1:**
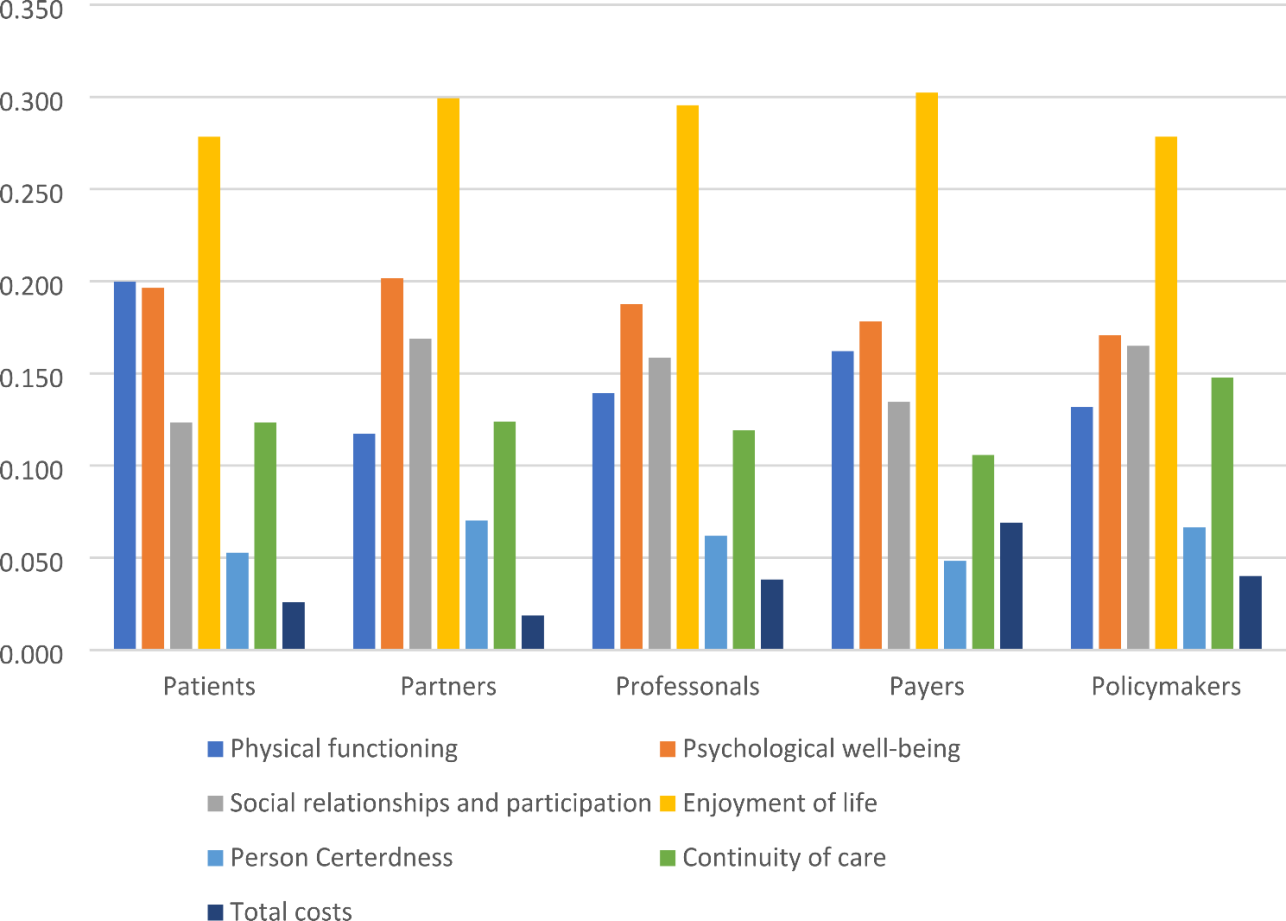
Relative preferences of different stakeholders across different outcomes

Relative weights for the different outcomes were gathered in an online study among Norwegian patients, informal caregivers, professional care providers, payers, and policy makers between 2017 and 2018, using a discrete choice experiment (DCE). Figure 1 illustrates the relative preferences of the outcomes included in the MCDA. All five stakeholder groups put relatively high weights on enjoyment of life and the lowest weight on cost (see [21] for detail).

### Statistical analysis of performance scores

To reduce systematic differences in the empirical distribution of the baseline confounders, and to minimize the effect of any potential selection bias at baseline between MAR-Bergen and SoC we performed *inverse probability of treatment weighting* (IPTW), using the propensity score [22–24].

Logit regression was used to estimate the propensity score in which treatment group (MAR-Bergen or SoC) was regressed on selected observed baseline characteristics: cohabiting status (living alone/ living with partner/ living with others) and financial condition/independence, (age and gender were not included as they were considered for defining subgroups in constructing the pseudo-panel dataset), and the baseline values of five outcome variables, which were physical health, social relationships and participation, person centeredness, continuity of care, and cost. In the IPTW the weights of the patients in MAR-Bergen were set at 1 and the weights of the MAR-Oslo patient-cohorts were calculated with the formula

$$Weight = {\text{ }}propensity{\text{ }}score/\left( {1 - propensity{\text{ }}score} \right).$$

The baseline (2017) differences between the MAR-Bergen and SoC cohorts were assessed before and after the IPTW. Overall matching results were assessed by examining and reporting three test statistics: the mean (median) absolute standardized bias; Rubin’s B, and Rubin’s R. Rubin suggested the value of B should be less than 25 and R between 0.5 and 2 for sufficient balance [25]. Statistical significance of the standardized differences has also been presented for all covariates before and after matching.

In estimating causal effects, we used a quasi-experimental impact evaluation approach and estimated the *average treatment effect on the treated* for all outcome measures on the IPTW data. Moreover, to address missing data for the groups in 2019, we adopted a robust solution—the mixed method approach—preserving the integrity of the Intent-to-Treat (ITT) principle [26]. We used models assuming continuous outcomes for ease of interpretation. Formally, we estimated the following equation:

$$\:{{\text{y}}_{jt}} = \beta {\:_0} + \beta {\:_1}{B_j} + \beta {\:_2}{T_t} + \beta {\:_3}{B_j} \times \:{T_t} + \chi \:{X_{jt}} + {\nu _j} + {\varepsilon _{jt}}$$

where $\:{y}_{jt}\:$is the outcome for cohort j at time t; *Bj* is a dummy variable, where *Bj* = 1 if the cohort is from the MAR-Bergen (*Bj* = 0 if the cohort is from the SoC); and *T*_*t*_ is a dummy variable for time (equals 1 if the cohort is from 2019, equals 0 if the cohort is from 2017), respectively. The coefficient for $\:{\widehat{\beta\:}}_{3}$ describes the treatment effect. $\:{X}_{jt}$ includes cohort j^th^ average age at period t. $\:{\nu _j}$ is the random error term for the j^th^ cohort, and $\:{\varepsilon _{jt}}$ is the remaining error term for j^th^ cohort observed in the t^th^ period.

As a next step of an MCDA, to calculate **performance scores** on the outcome measures (the performance score typically denotes the score on a single outcome, reflecting the partial score between alternative programs, such as intervention vs. comparator), we predicted the mean score of the cohort from MAR-Bergen in 2019 based on the results of the regression. In addition, we predicted the mean score of the SoC patient-cohorts assuming they had the same baseline (2017) score as the MAR-Bergen cohorts. This would tell us how much better (or worse) a patient subgroup would do if they were treated in MAR-Bergen relative to SoC. In this way, the calculated performance scores could be directly compared between the groups. This was done separately for each outcome.

### Overall value calculation

As to follow the next step of an MCDA, the mean predicted outcome scores at T_1_ (2019) were first standardized on a 0–1 scale. This was done using relative standardization with the equation:$\:{\:S}_{aj}=\frac{{x}_{bj}}{{\left({x}_{bj}^{2}+{x}_{oj}^{2}\right)}^{1/2}}$, where $\:\:{x_{bj}}$ is the raw performance score in terms of mean predicted values for outcome j (on the natural scale) for the MAR-Bergen group and;$\:\:{x}_{bj}^{2}\:and\:{x}_{oj}^{2}\:$ are the square of mean predicted values for outcome j for the MAR-Bergen and the SoC respectively. For all outcomes, the *standardized performance score* was set so that a higher score indicates better performance. To achieve this, in the above-mentioned equation, $\:{x}_{bj}^{2}\:and\:{x}_{oj}^{2}$ were replaced by $\:\frac{1}{{x}_{bj}^{2}}and\:\frac{1}{{x}_{oj}^{2}}$ respectively for the outcome—*Total costs*—where a higher score on the natural scale indicates worse performance. To combine preference weights and performance scores of each outcome into an *overall value score*, we implemented an additive MCDA model and used a weighted sum approach:

$$\:Overall\:Value\:Scor{e_i} = \:\sum {Weigh{t_{io}}*Normalized\:performance\:scor{e_o}} \:$$

where *i* indicates stakeholders that ranges between 1 and 5, and *o* represents outcome that ranges between 1 and 7 outcome measures.

### Sensitivity analysis

To perform the final MCDA step, probabilistic sensitivity analysis using Monte Carlo simulation was performed to evaluate the joint uncertainty of preference weights and performance scores. Cholesky decomposition was conducted for 10,000 replications to obtain a single overall value score. We calculated confidence (uncertainty) intervals around the overall value scores for each stakeholder group. The difference between the overall value score of the MAR-Bergen and SoC is statistically significant if the confidence intervals do not overlap.

All statistical analyses were performed with STATA 16.1.

## Results

### Sample characteristics

The background characteristics of the sample are presented in Table 2. Before the IPTW, the average age of the cohort with opioid dependence at baseline was around 44 years for the MAR-Bergen group, while the corresponding average age was higher for the SoC group, at 46 years, though the difference was not statistically significant. Regarding group differences in the baseline values of all the outcomes measures— physical health, mental health, social relationships and participation, enjoyment of life, person centeredness, continuity of care and total cost - after the IPTW all p-values for the standard differences were found insignificant. Overall, as illustrated in the IPTW statistics given in the lower panel of Table 2, the IPTW led to an improvement in the comparability of the two groups. The statistics satisfied the required criteria with recommended ranges. Mean (median) standardized bias was 30.4 (27.1) in the unmatched and without the IPTW sample, and after implementing the IPTW using the propensity score approach in the IPTW sample it was reduced to 7.6 (5.3); Rubin’s B was 90.1 in the unmatched and unweighted sample and reduced to 24.9 for the IPTW sample, and Rubin’s R was 1.24 in the unmatched and unweighted sample and slightly reduced to 1.22 in the IPTW sample.

**Table 2:** Baseline (2017) characteristics before and after the inverse probability of treatment weighting (IPTW) approach

Variable	Before IPTW			After IPTW	
	MAR-Bergen *N* = 90	SoC *N* = 101	*p*-value for the St. difference	SoC *N* = 101	*p*-value for the St. difference
	Mean (Std. Dev.)	Mean (Std. Dev.)		Mean (Std. Dev.)	
Age (in years)	43.63 (13.34)	46.07 (15.62)	0.250	47.15 (16.78)	0.237
Male	0.511 (0.503)	0.535 (0.501)	0.568	0.461 (0.501)	0.568
Live alone^#^	0.721 (0.250)	0.773 (0.264)	0.746	0.752 (0.289)	0.544
Live with partner^#^	0.132 (0.183)	0.098 (0.202)	0.162	0.128 (0.247)	0.906
Live with others^#^	0.169 (0.197)	0.140 (0.203)	0.216	0.145 (0.195)	0.481
Financial conditions/ independence^#,§^	2.350 (0.437)	2.217 (0.480)	0.317	2.280 (0.451)	0.364
Physical health^#^	2.288 (0.410)	2.178 (0.477)	0.089	2.267 (0.459)	0.789
Mental health	2.140 (0.363)	2.068 (0.4269	0.209	2.165 (0.394)	0.726
Social relationships & participation^#^	4.378 (0.681)	4.083 (0.621)	0.002	4.353 (0.725)	0.867
Enjoyment of life	1.850 (0.482)	1.876 (0.373)	0.679	1.982 (0.421)	0.105
Person Centeredness^#^	0.208 (0.218)	0.150 (0.211)	0.062	0.185 ((0.249)	0.629
Continuity of care^#^	0.690 (0.265)	0.562 (0.303)	0.002	0.694 (0.306)	0.933
Total Cost^#^	270.5 (55.96)	298.4 (58.71	0.045	273.5 (58.06)	0.775
**Statistics to assess propensity score-basedIPTW**					
Mean (Median) Bias^#^	30.4 (27.1)			7.6 (5.3)	
Rubin’s B^#^	90.1			24.9	
Rubin’s R^#^	1.24			1.22	

Note: ^#^ Variables used in propensity score; St. difference = Absolute Standardized Mean Difference, also called Absolute Standardized Bias

^§^The variable is defined (scored) as to whether a person: (i) has reasonably good order in the economy, based on a fixed income from paid work or permanent social security, possibly supplemented with financial social assistance or other temporary benefits (3); (ii) has some order in the economy, based on periods of wage income or benefits, but relatively dependent on financial social assistance or other temporary benefits or may have some illegal income (2); (iii) has a large disorder in the economy and/or is completely dependent on public financial benefits and/or illegal income (1)

### Effects of treatment on performance scores

The results of the statistical analyses of all seven outcomes included in the MCDA are represented in Table 3. The estimated treatment effects on the treated, $\:{\widehat{{\upbeta\:}}}_{3}$ (*coefficients of the interaction term between time and intervention*), are provided in the last column of Table 3. Both programs exhibited deterioration in various outcomes numerically MAR-Bergen demonstrated less deterioration than SoC though the differences were not statistically significant at a 0.05 alpha level.

**Table 3:** Health/Wellbeing and experience Outcomes^§^: estimates describing within- and between-group differences after 2 years

Outcomes included in the MCDA (*N* = 368)	Estimated change MAR-Bergen Mean	Estimated change SoC Mean	Diff. in change Mean (95% CI) ^#^
*Health/wellbeing*			
Physical functioning	-0.105	-0.096	0.009 (-0.114; 0.133)
Psychological well-being	-0.220	-0.223	0.003 (-0.151; 0.156)
Social relations & participation	-0.401	-0.436	0.035 (-0.284; 0.354)
Enjoyment of life	-0.104	-0.158	0.055 (-0.105; 0.215)
*Experience of care*			
Person-centeredness	-0.002	0.042	-0.044 (-0.182; 0.093)
Continuity of care	-0.077	-0.122	0.045 (-0.091; 0.181)
*Cost*			
Health and social care cost (a composite index)	33.415	37.299	-3.884 (-28.17; 20.40)

Note: ^*§*^ A negative sign indicates a deterioration for all outcomes except cost. A positive sign for cost variables implies a deterioration in health and social care costs expressed as a composite indicator (Table 1 illustrates the outcomes scale range)

^#^Based on robust standard error

### MCDA standardized performance scores and overall value scores

Table 4 reports the standardized performance scores and the overall value score in the multi-criteria decision analysis (MCDA) for five Norwegian stakeholder groups (5Ps): Patients, Partners, Professionals, Payers, and Policymakers. We found comparable standardized performance scores between both delivery models for physical functioning, psychological well-being, social relationships and participation, enjoyment of life, and total costs (2nd & 3rd column of Table 4). The MAR-Bergen program scored numerically higher on continuity of care (0.733 versus. 0.680), while the SoC-program scored higher on person-centeredness (0.772 versus 0.635). For all stakeholder groups, MAR-Bergen performed marginally better than SoC (e.g., 0.708 vs. 0.705 for patients). This small difference occurs because improvements in some outcomes (especially continuity) are offset by the deterioration in person-centeredness. The difference in scores between the two programs was similar for the five stakeholder groups.

**Table 4:** Stakeholders’ value scores in the Multi-criteria decision analysis

	Standardized performance score		Patients		Partners		Professionals		Payers		Policy makers	
			Weighted score		Weighted score		Weighted score		Weighted score		Weighted score	
	MAR-Bergen	SoC	MAR-Bergen	SoC	MAR-Bergen	SoC	MAR-Bergen	SoC	MAR-Bergen	SoC	MAR-Bergen	SoC
Health/Well-being												
Physical functioning	0.709	0.706	0.141	0.141	0.083	0.083	0.099	0.098	0.115	0.114	0.093	0.093
Psychological well-being	0.708	0.707	0.139	0.139	0.143	0.143	0.133	0.133	0.126	0.126	0.121	0.121
Social relationships & participation	0.710	0.704	0.088	0.087	0.120	0.119	0.113	0.112	0.096	0.095	0.117	0.116
Enjoyment of life	0.710	0.704	0.198	0.197	0.213	0.211	0.210	0.208	0.215	0.213	0.198	0.196
Person-centeredness	0.635	0.772	0.033	0.041	0.045	0.054	0.039	0.048	0.031	0.037	0.042	0.051
Continuity of care	0.733	0.680	0.090	0.084	0.090	0.084	0.087	0.081	0.078	0.072	0.108	0.100
Total costs	0.712	0.703	0.018	0.018	0.013	0.013	0.027	0.027	0.049	0.048	0.029	0.028
Over-all value score			0.708	0.705	0.707	0.706	0.708	0.706	0.708	0.705	0.708	0.706
Overall mean value scores including uncertainty [95% CI]			0.7109 [0.7107; 0.7112]	0.7013 [0.7012; 0.7016]	0.7101 [0.7099; 0.7103]	0.7019 [0.7017; 0.7021]	0.7105 [0.7103; 0.7107]	0.7016 [0.7014; 0.7020]	0.7105 [0.7103; 0.7107]	0.7006 [0.7004; 0.7008]	0.7115 [0.7113; 0.7117]	0.7018 [07017; 0.7021]
Percentage MAR-Bergen > SoC			67		63		65		69		65	

Relative weights for the different outcomes among stakeholders were elicited in an online weight elicitation study among Norwegian patients, informal caregivers, professional care providers, payers and policymakers. Figure 1 shows the relative weights of the outcomes included (i.e., Physical Health, Psychological well-being, Social relations & participation, Enjoyment of life, Person Centeredness, Continuity of care, and Total costs) in the multi-criteria decision analysis (MCDA) and as shown in the figure, all five stakeholder groups put relatively high weights on the Enjoyment of life (yellow) and the lowest weight on Total costs (dark blue)

### Sensitivity analysis

The robustness of the results was investigated by considering new sets of coefficients for treatment effect and importance weights using the Cholesky decomposition approach. The lower panel of Table 4 illustrates the uncertainty around the overall value score calculated with Monte Carlo simulation. For all five stakeholders, the MCDA results were very similar to our base case analyses, indicating that the overall value scores were higher for MAR-Bergen than SoC. The proportion of the 10,000 simulations showing a higher score for the integrated MAR-Bergen approach was between 63% and 69% for all stakeholders. The 95% confidence intervals *did not overlap.*

## Discussion

The study aimed to evaluate the comparative effectiveness of two distinct medically assisted rehabilitation (MAR) programs: the integrated MAR-Bergen program and the standard of care (SoC) for patients with opioid dependence in Norway, using the MCDA framework. This study showed that compared to the standard SoC approach, integrated MAR-Bergen treatment improves multiple outcomes for patients with opioid dependence; however, comparative improvements in the outcomes were not statistically significant. Moreover, there was an observed absolute deterioration in outcome levels in both the integrated MAR-Bergen and SoC treatment groups after follow-up. The result might suggest that more severe opioid patients were enrolled in both programs during follow-up, and/or that the short-run effects of the MAR patients were deteriorating for both programs.

Our MCDA results demonstrated that the standardized performance scores of the integrated tertiary care model (MAR-Bergen) were slightly better for physical functioning, psychological well-being, social relationships & participation, enjoyment of life, and total costs, whereas the person-centeredness outcome favors the primary care-oriented model (SoC). Additionally, MAR-Bergen demonstrated higher *overall value scores* compared to SoC for all five stakeholders.

Although, the standardized performance score for person-centeredness was higher for SoC, the continuity of care was higher for the MAR-Bergen program, the relative ‘weighted value scores’ were comparatively quite higher for continuity of care than person-centeredness for all five stakeholders suggesting that society puts more value on continuity of care than person-centeredness. Thus, continuity of care contributed to a higher overall value score of treatment which should advise when social gains of additional input would be most valued.

To our knowledge, this is the first MCDA study on people with opioid use dependence, particularly on the MCDA evaluation of medically assisted opioid substitution treatment programs. Thus, it is not possible to compare the findings with other studies using such a comprehensive approach. While our results indicated statistically non-significant effectiveness of the MAR-Bergen program, it is important to note that this might potentially be due to a limited sample size. It is crucial to recognize that ‘non-significance’ (e.g., *p* > 0.05) should not automatically imply the absence of a difference or the ineffectiveness of the intervention. Common issues such as a smaller-than-expected sample size, increased variability, or a lower incidence of outcomes can reduce statistical power, making non-significant results more likely, even when there may be a real and significant treatment effect. Nevertheless, previous literature showed that the effectiveness of integrated treatment in managing opioid dependence and interconnected services into the healthcare system enables healthcare providers to detect substance abuse at an early stage [5, 6]. Early identification of substance abuse allows healthcare providers to employ various intervention techniques, including motivational interviewing, which is essential for eliciting behavior change in patients with mixed feelings about altering their behavior. Patients in recovery may experience improved treatment retention, leading to reduced relapses and enhanced overall health outcomes.

The relevant literature widely demonstrates the benefits of integrated treatment, particularly in terms of relapse prevention and long-term outcomes. The integration of care may have both short-run and long-run effects. Over time, integrated care becomes not only more practical for people with opioid dependence but also serves to educate healthcare providers and counselors about their unique therapeutic tools and expertise, facilitating the adoption of medication and behavioral therapies that may have otherwise been rejected [29–30. We have acknowledged the challenge posed by the nature of studying a hard-to-reach population, which makes it difficult to attain a long-term longitudinal dataset with comparable data on both groups.

### Strengths and limitations

The integrated program MAR-Bergen includes patients with chronic health conditions and multi-morbidities. BrukerPlan register provides information on several domains of life for the relevant patient groups. Thus, using the MCDA framework this study showed that on certain outcomes MAR-Bergen marginally performed better than SoC, while on others it did not. Even though we are aware of the methodological challenges of the MCDA (for details see [9]), the study provides some evidence that traditional cost-effectiveness analyses may miss important features of relevant outcome space [21]. MCDA allowed us to use an explicit framework to aggregate the various outcomes and to calculate an overall value score. Moreover, we showed overall value scores from the perspectives of different stakeholders. The latter is important because making transparent the preferences of different stakeholders is likely to contribute to the political processes of decisions making and implementation of integrated care programs, in particular, for vulnerable groups like the patient group studied here. There is good reason to have confidence in the weights attached to the outcomes, obtained in a discrete choice experiment among a total of 776 stakeholders (~ 150 per stakeholder group) [21].

The performance scores were estimated using a quasi-experimental framework by pseudo-longitudinal data. This innovative approach enables us to construct a control cohort, and follow the cohorts, which will provide better estimates of the impact of integrated programs. Moreover, the uncertainty around the overall value scores was formally incorporated using probabilistic sensitivity Monte-Carlo simulations on both weights and scores. To our knowledge, this is rare in this field of study. Incorporating the uncertainty around the overall value scores using probabilistic sensitivity Monte-Carlo simulations on both weights and scores further strengthens the study.

There are some methodological limitations with the MCDA framework discussed in the literature [see 9–10, 27, for detail]. Though the SELFIE-MCDA framework has made efforts to overcome several challenges facing the method (see [15]). This study used two outcomes definitions–one for the performance and the other for the weight measurement. The question could be raised whether and to what extent the mapping of the outcome variables was adequate. Moreover, our data is approximately five years old, but we assume that the situation remains relatively similar. Furthermore, lack of data on the volume of health and care services used by the users implies that we had to rely on a composite indicator to proxy the total health and care cost.

The lack of other relevant covariates (e.g., clinical features, multimorbidity status), in our data implies that we were not able to include these variables in the compilation of the propensity score. We acknowledge the inherent limitation that we cannot empirically test exchangeability [28]. The nature of a study on a hard-to-reach population, makes it difficult to achieve a long-term longitudinal data set with comparable data on both groups. We recognized that the non-significant results between MAR-Bergen and SoC could be due to chance, lack of power, or an absence of true program benefit. It is challenging to disentangle the possibilities. Another potential factor that could contribute to this insignificant finding is if the exposure period for patients included in MAR-Bergen is shorter than for those in SoC.

Our study is constrained by the absence of information regarding the timing of patient exposure to the MAR-Bergen and SoC programs within our dataset. Our understanding is that patients in Bergen with opioid dependence received the MAR-Bergen treatment approach, while patients in Oslo received the standard of care (SoC) treatment during our study period. The validity of the assumption may be questioned. However, ethical considerations aimed at protecting the identity and personal information of subjects limited our dataset in this regard. We assume that the exposure period for patients enrolled in the two alternative programs/set-ups is random, implying no systematic difference in the average timing of exposure between the intervention and comparison groups. Cautious conclusions are warranted regarding the possibility that people with opioid dependence may not have received treatment for a long enough duration to fully benefit from the integrated program.

## Conclusions

The study is a novel contribution to the research into a problem which is high on the political agenda. Acknowledging the significance of different life aspects, including the “Triple Aim”, the study emphasizes the need for integrated care for the people with opioid dependence.

We conclude that the two highly effective MAR treatment approaches yield promising outcomes with a challenging population and are quite similar. It is possible that the ‘integrated care’ apparatuses are not so efficient, or that SoC already includes some integrated care components, limiting the added value of the MAR-Bergen program. The long-run effects the integrated care may be more impactful than short-run ones. Therefore, we strongly recommend further exploration of the nuances of each approach through a more rigorous controlled design with long-term longitudinal data. It would also be valuable to investigate how the positive aspects of both approaches can be combined to optimize outcomes.

## Electronic supplementary material

Below is the link to the electronic supplementary material.

Supplementary Material 1

## Data Availability

The data analyzed in the study was gathered by the Centre for Alcohol and Drug Research (KORFOR), Stavanger University Hospital (SUS), Norway and their use has been approved by the Regional Committee for Medical and Health Research Ethics (reference number 0181/2131/ REK west, dated 12.4.2019). According to Norwegian legislation data cannot be made available for use beyond what has been approved by the ethical review board. Therefore, the data cannot be made publicly available request due to privacy/ethical restrictions. Data are however available from the last author (JEA) upon reasonable request and with permission of the Centre for Alcohol and Drug Research (KORFOR), Stavanger University Hospital (SUS), and the Regional Committee for Medical and Health Research Ethics, Norway (https://www.forskningsetikk.no/en/about-us/our-committees-and-commission/rek/).
