# Supplementary material for: Medically assisted integrated rehabilitation program for people with opioid dependence: a quasi-experimental evaluation using multi-criteria decision analysis
Source: BMC Psychiatry. 2024 Dec 23;24:941. doi: 10.1186/s12888-024-06416-5 (PMC11667878; doi:10.1186/s12888-024-06416-5)
Supplement: Supplementary file 1 — Supplementary Material 1 [file 12888_2024_6416_MOESM1_ESM.docx]

**Appendix**

**Table A1: Mapping SELFIE Questionnaire with the outcome variables gathered from BrukerPlan data**

| SELFIE | BrukerPlan |
| --- | --- |
| **A. Physical functioning**  Does your health now limit you  in these activities? If so, how much?  A1 Vigorous activities, such as running, lifting heavy objects, participating in strenuous sports  A2 Moderate activities, such as moving a table, pushing a vacuum cleaner, bowling, or playing golf  A3 Lifting or carrying groceries  A4 Climbing several flights of stairs  A5 Climbing one flight of stairs  A6 Bending, kneeling, or stooping  A7 Walking more than a mile  A8 Walking several blocks  A9 Walking one block  A10 Bathing or dressing yourself  Yes, limited a lot  Yes, limited a little  No, not limited at all | Physical health in three categories: Green/Yellow/Red  Green: Does not have physical health problems with serious consequences for daily functioning.  Yellow: Has some physical health problems that have serious consequences for daily functioning and / or for future health.  Red: Has extensive physical health problems that have very serious consequences for the poor functioning and / or for future health condition. |
| **B. How you feel/mental health**  How much of the time during the past month?  B1 Were you a happy person?  B2 Have you felt calm and peaceful?  B3 Have you been a very nervous person?  B4 Have you felt downhearted and blue?  B5 Have you felt so down in the dumps that nothing could cheer you up?  All of the time  Most of the time  A good bit of the time  Some of the time  A little of the time  None of the time | Mental health in three categories: Green/Yellow/Red  Green: Mental health condition without any serious consequences for functional level and / or relationship with others.  Yellow: Some functional impairment due to mental health condition, fails to meet normal requirements for functioning towards friends, work / school, agreements, public transport, but reasonably takes care of their own daily chores and personal hygiene. (2)  Red: Severe dysfunction and failing forces, motivation and/or skills in relation to demands from the environment and for self-care (for example, daily chores and personal hygiene) because of the mental health condition. |
| **C. Enjoyment of life**   I can have all of the enjoyment and pleasure that I want   I can have a lot of the enjoyment and pleasure that I want   I can have a little of the enjoyment and pleasure that I want   I cannot have any of the enjoyment and pleasure that I want | Whether have an engagement in meaningful activity e.g. in work, education, or other activities:  in three categories: Green/Yellow/Red  Green: with sufficient degree.  Yellow: in some but not sufficient degree.  Red: very little or no meaningful activity. |
| **D. Social relationships and participation**  D1. My chances of talking to people close to me on equal terms are  D2. The quality of my relationships with people who are close to me are  D3. The respect I receive from people who are close to me is  D4. My relationships with acquaintances are  D5. The respect I receive from acquaintances is  D6. My chances of having an intimate relationship are  D7. My chances of seeing people as often as I want are  Very good  Good  Fair  Poor  Very poor | Relationships with social networks in three categories: -Green/Yellow/Red  Green: Has regular relationships with social networks such as family, children, friends, voluntary organizations and/or working life.  Yellow: Has limited relationships with social networks such as family, children, friends, voluntary organizations and/or working life.  Red: Has little or no contact with social networks of all kinds, are isolated or have only marginal social relationships  Social functioning in three categories: Green/Yellow/Red  Green: Have good social functioning to able to manage the daily chores.  Yellow: Limited social functioning to able to a limited extent to take care of the daily chores in homes, shops and in contact with public offices.  Red: Has very poor functioning in all types of contexts. |
| **E. Resilience**  E1. I tend to bounce back quickly after hard times  E2. I have a hard time making it through stressful events  E3. It does not take me long to recover from a stressful event  E4. It is hard for me to snap back when something bad happens  E5. I usually come through difficult times with little trouble  E6. I tend to take a long time to get over set-backs in my life  Strongly disagree  Disagree  Neutral  Agree  Strongly agree | Not available |
| **H. Person-centeredness**  H1. Did you discuss what was most important for YOU in managing your own health and well-being?  H2. Were you involved as much as you wanted to be in decisions about your care?  H3. Were you considered as a ‘whole person’ rather than just a disease/condition in relation to your care?  H4. Did your care team / providers involve your family/friends/carers as much as you wanted them to be in decisions about your care?  H5. Have you had enough support from your care team / providers to help YOU to manage your own health and well-being?  H6. To what extent did you receive useful information at the time you needed it to help you manage your health and well-being?  Not at all  To some extent  More often than not  Always | Whether have an individual plan with patient or patient relatives?  --Yes/no |
| **I. Continuity of care**  I1. My care providers transfer information very well to one-another  I2. My care providers work together very well  I3. My care providers are very well connected  I4. My care providers always know what one-another is doing  I5. I have to wait too long for an appointment or treatment  Strongly agree  Agree  Neutral  Disagree  Strongly disagree  N/A | Whether individual have a plan with:  general practitioner (GP)  or health and social care service provider or  plan with NAV or  plan with specialist care provider or plan with PPTOT or plan with child welfare:  --Yes/no |
| **J. Total Health and social care cost**  During the last 3 months, did you visit  J1. ……..your general practitioner?  J2. ……..a primary care nurse (e.g., practice nurse, nurse practitioner)  J3. ……..a GP assistant?  J4. ……..a physiotherapist?  J5. ……..an occupational therapist?  J6. ……..a speech therapist?  J7. ……..a dietician?  J8. ……..a podiatrist?  J9. ……..a medical specialist (e.g., geriatrician, lung specialist, cardiologist, eye specialist, rheumatologist, neurologist, elderly care physician)  J10. ……..a psychologist, psychiatrist or psychotherapist  J11. ……..a district nurse or community nurse?  J12. ……..a social worker?  J13. ……..a welfare worker?  J14. ……..a homoeopathist or an acupuncturist?  J15. ……..dentist?  J16. ……..optician?  □ No  □ Yes, namely X visit(s)  During the last 3 months did you visit any of the following services below, and if so, how often? Please only fill in day-visits (outpatient), and not overnight stays (inpatient)  J20. ……..residential care or nursing home  J21. ……..rehabilitation centre  □ No  □ Yes, namely X visit(s)  J23. During the last 3 months, did you visit a hospital emergency room?  J24. Have you been admitted to a hospital in the past 3 months?  J25. Were you admitted elsewhere because of your health during the last 3 months?  J26. ……..residential care or nursing home  J27. ……..rehabilitation center  J28. ……..psychiatric hospital  J29. Did you receive home care in the last 3 months?  J30. ……..housekeeping and home help  J31. ……..personal care  J32. ……..nursing  J33. Did you take any medication during the last 3 months?  □ Yes  □ No  J34. What medication did you take during the last 3 months? | **Living conditions index:**  Health and social care cost were proxied by a *Living conditions index* which is a composite indicator contains the eight living conditions areas (housing, meaningful activities, economy, physical health, mental health, substance abuse, social functioning, network). The living conditions areas constitute a score where the red score has the highest points. The index comprises the summed of the points from these eight living condition features.  The score varies between 138 and 552. A higher score corresponds (assumed to be) to the higher needs for health and social care, and indeed the higher total cost. |
